# Survival among patients with severe high cervical spine injuries – a TraumaRegister DGU® database study

**DOI:** 10.1186/s13049-020-00820-y

**Published:** 2021-01-06

**Authors:** O. Kamp, O. Jansen, R. Lefering, M. Aach, C. Waydhas, M. Dudda, T. A. Schildhauer, U. Hamsen, the TraumaRegister DGU

**Affiliations:** 1 Department of Trauma, University Hospital Essen, Hand and Reconstructive, Surgery, University of Duisburg-Essen, Hufelandstraße 55, 45147 Essen, Germany; 2 Department of General and Trauma Surgery, BG University Hospital Bergmannsheil, Bochum, Germany; 3 Institute for Research in Operative Medicine (IFOM), University Witten/Herdecke, Cologne, Germany; 4 Department of Spinal Cord Injury, BG University Hospital Bergmannsheil, Bochum, Germany; 5 Medical Faculty, University of Duisburg-Essen, Duisburg, Germany; 6 Committee on Emergency Medicine, Intensive Care and Trauma Management (Section NIS) of the German Trauma Society (DGU), Munich, Germany

**Keywords:** Cervical spinal cord injury, Injury severity score (ISS), Abbreviated injury scale (AIS), Revised injury severity classification II (RISC II), Outcome, Trauma register

## Abstract

**Background:**

Trauma is a significant cause of death and impairment. The Abbreviated Injury Scale (AIS) differentiates the severity of trauma and is the basis for different trauma scores and prediction models. While the majority of patients do not survive injuries which are coded with an AIS 6, there are several patients with a severe high cervical spinal cord injury that could be discharged from hospital despite the prognosis of trauma scores. We estimate that the trauma scores and prediction models miscalculate these injuries. For this reason, we evaluated these findings in a larger control group.

**Methods:**

In a retrospective, multi-centre study, we used the data recorded in the TraumaRegister DGU® (TR-DGU) to select patients with a severe cervical spinal cord injury and an AIS of 3 to 6 between 2002 to 2015. We compared the estimated mortality rate according to the Revised Injury Severity Classification II (RISC II) score against the actual mortality rate for this group.

**Results:**

Six hundred and twelve patients (0.6%) sustained a severe cervical spinal cord injury with an AIS of 6. The mean age was 57.8 ± 21.8 years and 441 (72.3%) were male. 580 (98.6%) suffered a blunt trauma, 301 patients were injured in a car accident and 29 through attempted suicide. Out of the 612 patients, 391 (63.9%) died from their injury and 170 during the first 24 h. The group had a predicted mortality rate of 81.4%, but we observed an actual mortality rate of 63.9%.

**Conclusions:**

An AIS of 6 with a complete cord syndrome above C3 as documented in the TR-DGU is survivable if patients get to the hospital alive, at which point they show a survival rate of more than 35%. Compared to the mortality prognosis based on the RISC II score, they survived much more often than expected.

## Background

Over the recent decades, traumatic injuries have been a significant cause of death and disability. Worldwide almost 10% of all deaths are related to trauma, and it is the leading cause of death and disability of children and young people from 4 to 44 years old. Spinal cord injuries, particularly the cervical spinal cord, are rare in this group but are associated with significant disability or death [1, 2]. In the United States of America an annual incidence rate of 40 per million per year is estimated, whereas in Western Europe it is about 16 per million per year [3–6]. Among all trauma-related injuries, if survived, cervical spinal cord injuries are among those with the most life restricting injuries for trauma patients if survived. Stephan et al. [7] observed that 50% of all spinal cord injuries compromise the cervical spine. About 16% of all cervical spine injuries are at a level C3 or above and are classified with an Abbreviated Injury Scale (AIS) of 6 [8].

In order to describe and compare injuries clinicians and researchers frequently use injury scores, for example, the Injury Severity Score (ISS) or the New Injury Severity Score (NISS). Both are based on the Abbreviated Injury Scale (AIS) of the Association for the Advancement of Automotive Medicine (AAAM) [9, 10].

The AAAM was founded in 1957 to investigate and prevent traffic accidents with motorized vehicles. To describe the impact of these accidents on trauma patients, J.D. States first presented the AIS in 1969 at the Stapp car crash conference to describe the impact of these accidents on trauma patients [9, 11]. Since its first publication, the AIS has been used as the classification system for examining injuries in epidemiology and trauma research as well as for in-hospital evaluation. The AIS is an anatomically based, injury severity scoring system that classifies these injuries into nine anatomic regions, using a six-point ordinal scale. Researchers reduced these nine anatomic regions to only six body regions, to perform the ISS. In all versions of the scoring system, the latest from 2015 [8, 12, 13] the severity of all injuries are rated in a six-level severity scale from one being a minor injury, up to six for injuries that are thought to be untreatable or even not survivable [14]. As the AIS does not explain the patient overall injury severity for patients with multiple injuries, it is the basis of most of the injury severity scoring systems such as the ISS or NISS [15, 16]. The ISS ranges between 1 and a maximum of 75 points. It is calculated as the sum of the squared severity scores of the three most affected body regions (out of the six regions defined). An injury with an AIS of 6 will automatically result in an ISS or NISS of 75, irrespective of concurrent injuries. Researchers use the AIS coding for injury epidemiology, trauma research and in-hospital evaluation, however, not to fully describe outcome or survival of trauma patients. To describe outcome and survival of trauma patients the AIS coding in one of several items to form outcome prediction models which involve also age and physiology of the patient.

The Revised Injury Severity Classification II (RISC II) as a prognostic score uses the AIS. It is used as a model for risk of death estimation in severely injured patients based on 15 different items [17].

Although injuries with an AIS of 6 are usually considered as unsurvivable, or better, actually untreatable, researchers have reported survivors with a cervical spinal cord injury at C3 or above in publications, even though this injury is rare.

This study aims to describe the outcome and survival rate of patients with a life-threating spinal cord injury (AIS 6) above C3 in a large trauma population and to analyze their impact on mortality prediction models.

The present study is in line with the publication guidelines of the TraumaRegister DGU®, it is a TR-DGU registered project (ID 2016–015) and is also an ethics committee approved retrospective study (no. of approval 16–5731).

## Method

The TraumaRegister DGU® of the German Trauma Society (Deutsche Gesellschaft für Unfallchirurgie, DGU) was founded in 1993. The aim of this multi-centre database is a pseudonymised and standardised documentation of severely injured patients.

Data are collected prospectively in four consecutive time phases from the site of the accident until discharge from hospital: A) Pre-hospital phase, B) Emergency room and initial surgery, C) Intensive care unit and D) Discharge. The documentation includes detailed information on demographics, injury pattern, comorbidities, pre- and in-hospital management, course on intensive care unit, relevant laboratory findings including data on transfusion and outcome of each individual. The inclusion criterion is admission to hospital via emergency room with subsequent ICU/ICM care or reach the hospital with vital signs and die before admission to ICU.

The infrastructure for documentation, data management, and data analysis is provided by AUC - Academy for Trauma Surgery (AUC - Akademie der Unfallchirurgie GmbH), a company affiliated to the German Trauma Society. The scientific leadership is provided by the Committee on Emergency Medicine, Intensive Care and Trauma Management (Section NIS) of the German Trauma Society. The participating hospitals submit their pseudonymised data into a central database via a web-based application. Scientific data analysis is approved according to a peer review procedure established by Section NIS. The participating hospitals are primarily located in Germany (90%), but a rising number of hospitals of other countries contribute data as well (at the moment from Austria, Belgium, China, Finland, Luxembourg, Slovenia, Switzerland, The Netherlands, and the United Arab Emirates). Currently, approximately 35,000 cases from almost 700 hospitals are entered into the database per year.

Participation in TraumaRegister DGU® is voluntary. For hospitals associated with TraumaNetzwerk DGU® however, the entry of at least a basic data set is obligatory for reasons of quality assurance.

In the present study, we analyzed the registry data from 2002 to 2015. The inclusion criteria were as follows: adult trauma patients with an age of 16 years or above and treated in a European trauma centre with a severe spinal cord injury (AIS 3). In order to avoid double-counting, we excluded patients transferred out within 48 h, as they appear as a transfer in the receiving hospitals.

The definition of cervical spinal cord injury is based on the Abbreviated Injury Scale, version 2005(updated 2008), which is used in the TraumaRegister DGU® (Table 1). An AIS 6 classifies patients with a cord contusion or laceration at C3 or above with a complete cord syndrome.

**Table 1:** Abbreviated Injury Scale of cSCI 2005(updated 2008) [12] and proportional distribution of injury severity for cSCI in TR-DGU out of 102,553 patients

AIS score	Description of injury	n	%
**3**	Transient neurological signs (paresthesia)	2245	2.2
**4**	Contusion with incomplete cord syndrome	1001	1.0
**5**	Contusion with complete cord syndrome C4 or below Laceration with complete cord syndrome C4 or below	1866	1.8
**6**	Contusion with complete cord syndrome C3 or above Laceration with complete cord syndrome C3 or above	612	0.6

### Statistical analysis

Data is presented as a number of cases with the percentage in case of categorical variables and mean with standard deviation in case of measurements. If the distribution of values were skewed, the median was presented alongside. Survivor and non-survivor results were compared using a chi-squared test or Mann-Whiney U-test, as appropriate. A *p*-value of < 0.05 was considered statistically significant. Were necessary we used the interquartile range (IQR). Statistical analysis was carried out using SPSS (Version 22, IBM, Armonk, NY, USA).

## Results

**Fig. 1:**
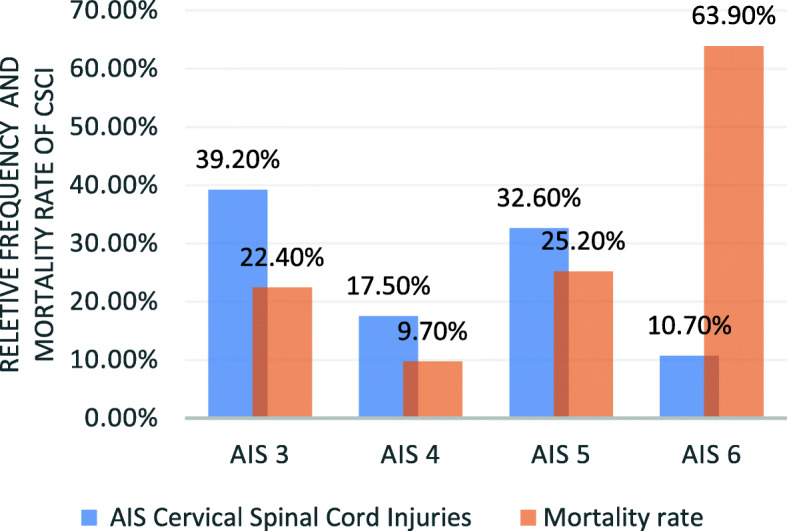
Relative Frequency and mortality rate of all entered cervical spinal cord injuries

From a total of 102,553 patients, we identified *N* = 5724 (5.6%) that had a severe cervical spinal cord injury (AIS 3 to 6). From the total population, 612 (0.6%) had a spinal cord injury AIS 6 and suffered from a spinal cord injury at C3 or above. Within the subgroup of patients with cervical spinal cord injury, the portion of SCI at C3 or above is as high as 10.7% (Fig. 1).

The mean age of the SCI 6 patients was 57.8 (SD 21.8) years. On average, patients who survived were 56.0 years old, and patients who died were 58.9 years old. 72% of all patients with cervical spinal cord injury were male, 28% were female.

Table 2 shows the demographic, physiological and clinical parameters of survivors and non-survivors.

**Table 2:** Demographic and clinical data comparing survivor and non-survivor for SCI AIS 6

	SCI 6 overall	SCI 6 non-survivor	SCI 6 survivor	*p*-value
Patient (n)	612	391 (63.9%)	221 (36.1%)	
Age (years)	57.8 (± 21.8)	58.9 (± 22.9)	56.0 (± 19.5)	0.020
Male (%)	441 (72%)	269 (69%)	172 (78%)	0.015
Pre-hospital GCS	4.5 (± 3.6)	3.5 (± 2.1)	6.8 (± 5.0)	< 0.001
Pre-hospital Cardio-pulmonary Resuscitation	327 (65%)	267 (78%)	60 (38%)	< 0.001
Pre-hospital heart rate in bpm	58 (± 47)	52 (± 51)	71 (± 36)	< 0.001
Pre-hospital systolic blood pressure mmHG	74 (± 61)	63 (± 61)	99 (± 53)	< 0.001
Heart rate on admission in bpm	83 (± 32)	84 (± 36)	80 (± 22)	< 0.001
systolic blood pressure on admission mmHG	102 (± 41)	96 (± 46)	114 (± 27)	< 0.001
Days of ventilation	10,9 (± 18.0) median 7	5.0 (± 7.8) median 4	21,2 (± 24,8) median 19	< 0.001
Days of ICU stay	12.4 (± 18.7) median 8	5.4 (± 8.4) median 4	24.7 (± 24.6) median 20	< 0.001
Days of hospital stay	20.2 (± 38.2) median 8	6.7 (± 11.4) median 3	44.0 (± 54.1) median 25	< 0.001

580 (98.6%) SCI 6 patients suffered a blunt trauma, and 8 (1.4%) presented a penetrating trauma. A total of 301 patients (49.2%) were injured in traffic accidents, while 29 patients (4.7%) were injured through attempted suicide. Regarding falls, 162 (28.9%) suffered from a low fall and 65 (11.6%) a high fall (> 3 m).

478 (78.1%) SCI 6 patients were treated in a level one trauma centre, either as primary admission (*n* = 375) or as transfer (*n* = 103). 122 (19.9%) AIS 6 patients were treated in a level 2 trauma centre.

327 (65%) required cardio-pulmonary resuscitation (CPR) on the scene. For the non-survivors, the CPR rate was 78% (267 out of 391 patients). 38% of the surviving patients (60 out of 221 patients) had CPR on the scene.

The onsite physiological parameter for systolic blood pressure for non-survivors was 63mmHG and for survivors 99mmHG. The heart rate for non-survivors was 52 bpm and for survivors 71 bpm.

**Fig. 2:**
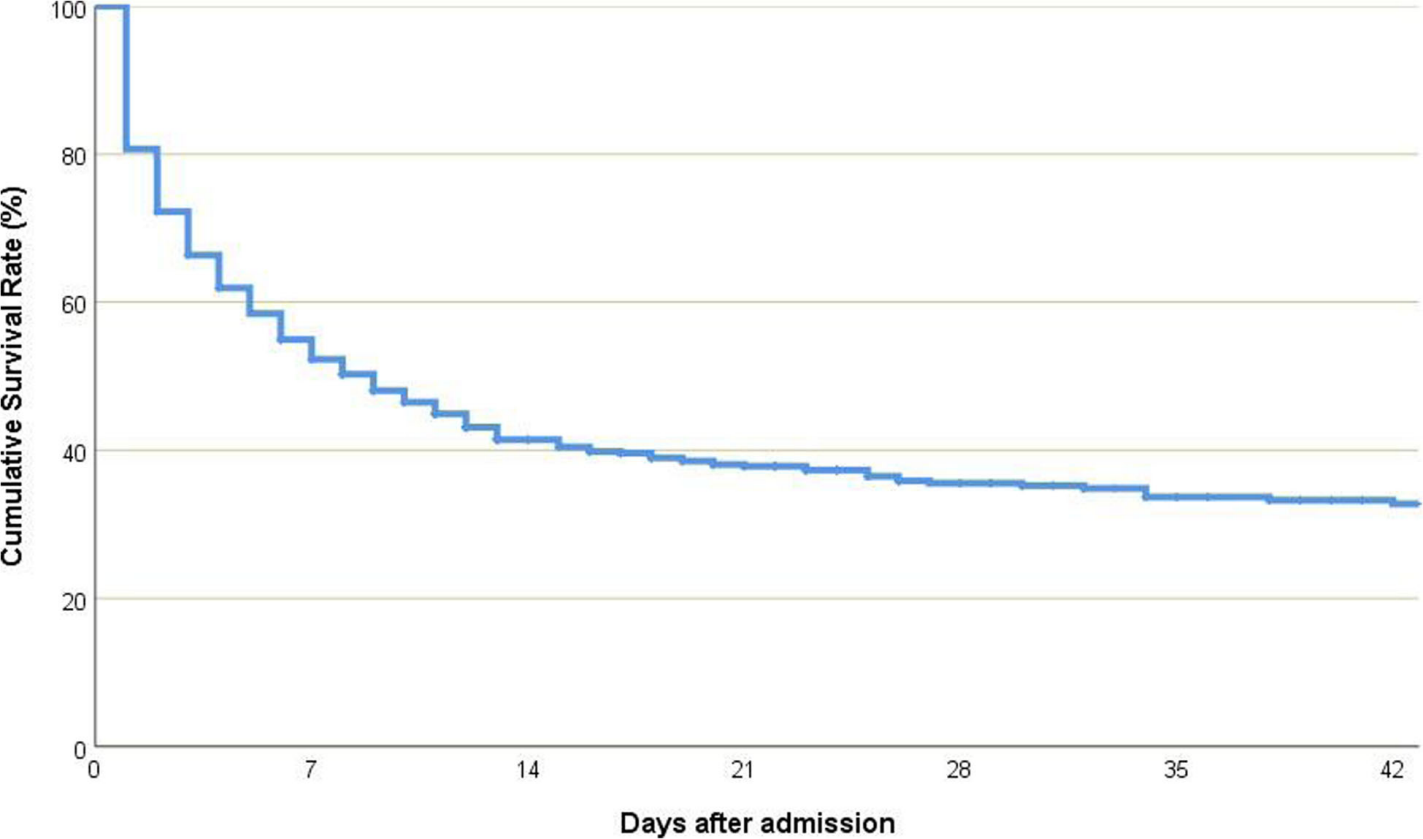
Kaplan-Meier survival curve for patients with severe cervical injury (AIS 6)

Out of the 612 included patients with cSCI and AIS 6, 391 (63.9%) died during their hospital stay. One hundred seventy patients (43.5%) died within 24 h after admission to hospital. The median of patients who died is 3 days (IQR 1–8 days) (Fig. 2). The RISC II Score showed a predicted mortality rate of 81.4% for all cSCI six patients. However the observed mortality rate for this group was 63.9%.

## Discussion

Traumatic cervical spinal cord injuries are one of the most life restraining injuries with a significant clinical and socioeconomic impact if survived [18]. In our study of severely injured patients, we could show that mortality prediction with the RISC II score for patients with a cervical spinal cord injury AIS 6 is highly overestimated with 81.4% as compared to the observed mortality rate of 63.9%. In contrast, for patients with a maximum AIS of 5 for cervical spinal cord injuries, the prediction (18.5%) is correct with an 18.5% observed mortality rate. There could be several reasons for this discrepancy such as the parameters which are included in the RISC II score, particularly the worst injury, age, motor function and physiological parameters which are represented by the ISS, GCS, CPR and blood pressure in the data of the TraumaRegister DGU®. We assume that these parameters, which all affect the outcome of spinal cord injuries and are all included in RISC II Score, could be a reason for the significant difference between the estimated and the real survival rate in this group of patients with a severe spinal cord injury.

The annual incidence of SCI’s ranges around the world. In the developed countries like the US it varies from 40 to 50 per million population, whereas in Europe the range estimate is reported to be between 13 and 19 per million population [3–6, 19–21]. For traumatic cervical spine injuries, the incidence was previously reported to be 16,5 per 100,000 hospital admissions in a Norwegian population [22]. Passias et.al [23]. showed that in 2017 the US population had an incident rate of 5.0% for traumatic cervical spine injuries. These previous findings are consistent with our overall incidence rate of 5.6% for all traumatic spine injuries in our data set.

The prevalent cause for spinal injuries in most studies are motor vehicle accidents (MVA) or falls. Passias et.al [23]. reported numbers for MVA and falls with 29.3 and 23.7%, which is relatively consistent with our study for MVA (35.5%) and falls (28.9%). The small variation seems to be due to our small sample size of 612 patients. Jackson et al. [24] showed a decrease for spinal cord injuries in the elderly in a study on the US Model Spinal Cord System, but other authors have shown that with increasing age there is an increasing risk for spinal cord injuries, especially for cervical injuries [25, 26]. Elderly patients often have a narrow spinal canal and a stiff spinal column, sometimes associated with Bechterews Disease, which could lead to cervical spinal injuries even with falls lower than 2 m [27]. In our population, 54% of all patients with cervical spinal cord injuries with an AIS of 6 were 60 years or older, whereas in the overall population only 36.5% were 60 years or older.

Guidelines for trauma care suggest that transferring patients with a cervical injury to a level 1 trauma centre could benefit them. In the study by Varma et al. [28] they could show, that 62% of all spinal cord injury patients were transferred to a level 1 centre. In our study, patients were transferred in 78.1% of all cases. However, as there are different health care systems and small sample sizes, these findings are difficult to compare. In the US, 84% of all citizens have access to level I or II trauma centre within 1 h, although over 46 million residents, mostly in rural areas, do not have one-hour access to level 1 or 2 trauma care [29]. In another country, Canada, it is estimated that about 22.5% of all residents do not have access to level I or II trauma care within 1 h [30]. Due to the existence of the TraumaNetzwerk DGU®, there is a trauma network with the potential of a level I or II trauma care within a 30 km radius. We assumed that this infrastructure could be a reason for the higher survival rate of patients with cervical spinal cord injuries in Germany.

Varma et al. [28] also observed that injuries of the cervical spine causes death at the accident site more often than other multiple injuries with cardiovascular instability. For patients with cervical spinal cord injuries, cardiovascular dysfunctions are initially the most life-threatening events, especially hypotension and bradycardia with resulting in an on-site CPR. Guly et al. [31] showed an incidence rate of 19% for hypotension and spinal shock of all patient with cervical spinal cord injury. According to Hagen et.al [32]. these findings result from injuries to the autonomic nervous system following a spinal cord injury. The injuries to the autonomic nervous system may also cause bradycardia which could be another cause for onsite CPR [31, 33]. Even the results are difficult to compare, in our group of patients the systolic blood pressure on scene was 63mmHG for the non-survivors and 99mmHG for the survivors. Regarding onsite CPR, in our group we observed that 78% of all non-survivors had a CPR, in contrast to 38% of all survivors. In contrast to our findings, a study from Lockey et.al [34]. showed that for 909 patients with traumatic cardiac arrest, the long-term survival rate with discharge from hospital was 7.5%. However, in this study they only had 6 (8.8%) cervical spine injuries out of 68 survivors. Lockey considered hypoxia as the main cause of traumatic cardiac arrest besides hypovolemia. In our study, we could only generate onsite oxygen saturation for 200 patients. Here we have to admit that based on the register style and the missing information we cannot draw conclusions about hypoxia in our study. In contrast to the American College of Surgeons and the National Association of EMS Physicians [35] we believe that based on our findings traumatic cardiac arrest from a cervical spinal cord injury should be resuscitated no matter of pulselessness or apneic events.

Varma et.al [28]. mentioned severe preexisting comorbidities are a main predictor for early death after trauma. In the TR-DGU only the ISS is reported, including all trauma related injuries, whereas the register does not report preexisting comorbidities. In case of our study we note that all patients with an AIS 6 injury are automatically set to an ISS of 75 due to the calculation method of the ISS [15]. Trauma patients with an ISS of 75 are often described as the most severe injuries with the lowest possible survival rate among trauma researchers. In several studies [36, 37] researchers excluded this type of patients. But Peng et al. [38] showed that among severely injured patients with an ISS of 75, 48.6% of all patients survived. We could confirm this in our study, as for patients with a spinal cord injury AIS of 6 saw a survival rate of 37.2%.

In our population the median stay on ICU for AIS 6 survivors was 20 days and in hospital stay was 44 days as mentioned in Table 2. Costa et.al [39]. laid out 2016 ventilator associated pneumonia is highly accompanied by physician staff and nurse work environment. As this is only one of many problems in the treatment of spinal cord injuries [40] with better equipment and highly trained staff the outcome gets better over time. We deem the implementation of spinal cord centers in Germany could also improve the survival and outcome of AIS 6 injuries.

The ISS has become the most cited and used trauma score in the last decades not only for trauma surgeons but also for researchers. But we have to note that the score disregards multiple injuries in the same body region and may underestimates head injuries [41, 42]. Paffrath et al. [43] asked whether the ISS based approach to severely injured patients is sufficient. They pointed out, that this approach on a purely anatomical background includes a major number of patients who are not at major risk to die. Here based on the register information of the TR-DGU Lefering et al. published the Revised Injury Severity Classification, version I and II [17, 44]. In the latest version of this trauma score 15 items, including anatomical and physiological parameter, were used out of the documentary of the TR-DGU to predict the mortality rate of each patient. Even with missing items in the register, the score is possible to calculate. As mentioned in the presentation of the RISC II, there are injuries which are overestimated with increasing risk of death. Here we could show that even the RISC II Score performs correctly for patients with a maximum spinal cord injury of AIS 5, but the group of cervical spinal cord injuries with an AIS 6 is highly overpredicted. Therefor in a revision of the RISC II there should be a reconsideration of the role of these AIS 6 injuries.

Furthermore, the ISS relies on the AIS codebook, which is repeatedly changed and updated. However, the revision in 2005 (update 2008) showed only major revisions for pelvic fractures, extremities and head trauma with almost no changes in spine, neck, abdomen and external injuries [45, 46]. Therefore, there were no changes that effected our study. For the years 2002–2008 the AUC performed a new coding with the 2008 codebook for all cases entered the TR-DGU, therefore no changes in count of cSCI are scarcely to be expected. Although there is a new revision in 2015, the AIS codebook of 2008 is still in use for the TR-DGU.

As well there were changes in coding in the revision 2005/08 there were also changes in the characterization of AIS 6 injuries. The wording changed from unsurvivable to not treatable. With this wording these injuries are better described.

We have to emphasize, that there are no injury scores or prediction models which weight all the different AIS 6 injuries of all body regions. Due to the small sample size, these injuries show no influence on the general quality of the prediction models.

As we have demonstrated above, we believe that neither changing the AIS codebook for cervical spinal cord injuries nor changing all mortality prediction models would be a solution, as these prediction models perform well in the majority of cases with exception of AIS 6 injuries. Therefor in a revision of the RISC II there should be a reconsideration of the role of the AIS 6 injuries.

Nonetheless in all clinical cases with an cSCI AIS 6 we speak for the treatment of these patients, because as we could show, there are more to survive than expected.

## Limitations

As a register-based study, there are some limitations involved in this analysis. First and foremost, the quality of register studies is inferior to clinical trials. Some vital information or parameters may be missing due to the register style. Case completeness can also be problematic and furthermore hospitals may not enter all their trauma patients into the TraumaRegister DGU®. We have to admit, that all registers depend on the correct classification of all injuries by the participating hospitals.

In addition, registers only include patients are who survived the admission to the hospital and in the case of our study, this could influence the outcome.

As a retrospective study, there are also some other facts to consider. The analysis is done after the event of interest had passed, which increases the likelihood of basic information and ascertainment bias. The injury severity can also be described with the American Spinal Injury Association (ASIA) classification, however only the AIS score is recorded in the data of the TraumaRegister DGU®.

## Conclusion

The Injury Severity Score is the basis for different trauma and mortality prediction scores as well as the RISC II score. Even though some authors describe an AIS 6 injury as not survivable, we show that there are more survivors than expected. Based on the RISC II score, we could show that only 63.9% died, whereas 81.4% were estimated. In contrast the predicted mortality for the overall population was correct with 18.5%. In the future this could potentially lead to changes in the role of AIS 6 injuries in mortality prediction models for this small sample size of patients.

## Data Availability

Apart from data included in the manuscript supporting the conclusions of this article, datasets of the TR-DGU are not publicly accessible. Data access requires permission by the Committee on Emergency Medicine, Intensive Care and Trauma Management (Sektion NIS) of the German Trauma Society.

## Abbreviations

AAAM: Association for the Advancement of Automotive Medicine
AIS: Abbreviated Injury Scale
AUC: Akademie der Unfallchirurgie
cSCI: Cervical spinal cord injury
CPR: Cardio-pulmonary resuscitation
GCS: Glasgow Coma Scale
ICM: Intermediate Care Unit
ICU: Intensive Care Unit
ISS: Injury Severity Score
MVA: Motor vehicle accidents
NISS: New Injury Severity Score
RISC II: Revised Injury Severity Classification II
TR-DGU: Trauma Register der Deutschen Gesellschaft für Unfallchirurgie
